# Dynamic nomogram for predicting long-term survival in patients with brain abscess

**DOI:** 10.1186/s41016-025-00402-w

**Published:** 2025-08-07

**Authors:** Thara Tunthanathip, Rakkrit Duangsoithong, Waranyu Kittirojkasem, Akira Pongweat, Rattiyaphon Khongthep, Benchamat Sutchai, Assama Tohyunuh

**Affiliations:** 1 Division of Neurosurgery, Department of Surgery, Faculty of Medicine, Prince of Songkla University, Songkhla, 90110 Thailand; 2 Department of Electrical Engineering, Faculty of Engineering, Prince of Songkla University, Songkhla, Thailand; 3 Faculty of Engineering, Prince of Songkla University, Songkhla, Thailand

**Keywords:** Brain abscess, Prognosis, Survival, Nomogram, Infection, Long-term survival

## Abstract

**Background:**

Brain abscess (BA) is a serious condition that causes significant mortality and morbidity. While various prognostic factors have been studied, there is limited research on long-term survival predictions. The present study aimed to identify predictors of long-term survival in BA patients and develop a dynamic nomogram for individualized prognostication. Additionally, the secondary objective was to develop and validate a dynamic nomogram for predicting long-term survival in BA patients.

**Methods:**

A retrospective cohort study was conducted on BA patients diagnosed at a tertiary care hospital in Southern Thailand. Demographic, clinical, laboratory, and imaging finding were analyzed. Cox regression was used to identify independent prognostic factors. A dynamic nomogram was developed and validated using Harrell’s concordance index (C-index), calibration plots, and cumulative case/dynamic control survival receiver operating characteristic (ROC) curves.

**Results:**

A total of 205 patients were included, with a mean follow-up of 41.66 months. The 1-year, 2-year, and 5-year survival probabilities were 0.77, 0.73, and 0.69, respectively. Independent predictors of long-term survival included age, Karnofsky performance status, hemoculture results, preoperative coagulopathy, neutrophil-to-lymphocyte ratio, bandemia, and occipital BA. The dynamic nomogram revealed strong predictive performance, with a C-index of 0.855 for apparent validation and 0.701 for validation with testing data. Calibration plots and ROC analysis further supported its reliability.

**Conclusions:**

This study presents a validated dynamic nomogram for predicting long-term survival in BA patients. The model provides an interactive tool for individualized risk assessment and facilitating clinical decision-making. Future research should focus on external validation and refinement of the model for broader applicability.

## Background

A brain abscess (BA) is a life-threatening disease caused by a localized infection of the brain parenchyma. Although there have been improvements in medical and surgical treatment, BA still causes mortality and functional disability, particularly in resource-limited settings [1, 2]. Brouwer et al. conducted a systematic review and meta-analysis of outcomes in patients with BA and found that mortality rate ranged from 10 to 40% [1]. A previous study by Ooi et al. found that the morbidity rate for pediatric BA patients in low- and middle-income countries was 6.9% and the mortality rate was 11.0% [2]. Additionally, the fatality rate for BA patients in Thailand was 27.2% [3], whereas the death rate for BA patients in Cameroon was 21.42% [4]. 

Predictors have been investigated from the review of literature. Advanced age, low Glasgow Coma Scale (GCS) score, immuno-compromised status, congenital heart disease, staphylococcal infection, low monocyte, hyponatremia, and hyperglycemia were reported associated with death [5–8]. However, a lack of research with long-term follow-up was noted. Bodilsen et al. reported on the prognosis of BA patients using survival analysis. As a result, the median follow-up time was 5.9 years, and the risks of mortality at 1-year, 2–5 years, and 6–30 years for BA patients were 21%, 16%, and 27%, respectively [9].

A clinical prediction tool (CPT) for long-term prognosis is essential for optimizing resource allocation, guiding clinical decision-making, and improving clinical outcomes [10]. For instance, the CPT might be a useful tool for identifying high-risk individuals and developing individualized treatment plans to reduce complications and improve survival [11]. Nomogram is one of the CPTs that has been investigated for prognostication in a variety of neurosurgical conditions such as traumatic brain injury [12], brain tumor [13], and surgical site infection [14]. Although nomogram is a simplified visual calculator for estimating the risk score for prognostication, the CPT’s development has been challenged by short-term follow-up data or a failure to incorporate dynamic factor such as clinical status or immunonutritional markers [15, 16]. When individual clinical conditions are altered, their prognosis and survival probability may change [15]. Therefore, a dynamic nomogram has been proposed in previous studies. In order to forecast the prognosis of patients with gastric cancer, Talebi et al. [17] suggested a dynamic nomogram based on the Cox regression model for prognostication in gastric cancer. They found that the tool’s Harrell’s concordance index was 0.64. Callegaro et al. [18] developed a dynamic nomogram for the purpose of prognostication in soft tissue sarcoma, and the tool’s concordance index ranged from 0.675 to 0.810.

In the literature review, there was a lack of evidence regarding the dynamic nomogram that provides long-term projections for BA patients. Therefore, the primary objective of the present study was to investigate the predictors associated with long-term prognosis of patients with BA. Additionally, the secondary objective was to develop and validate a dynamic nomogram for predicting long-term survival in BA patients.

## Methods

### Study design and study population

This is a retrospective cohort study of individuals who had a BA that required either pathological confirmation by a pathologist or neuroimaging plus intraoperative purulent findings at a tertiary care hospital in southern Thailand between January 2014 and August 2024. Exclusion criteria included incomplete medical records and unavailable preoperative neuroimaging. The neurosurgeon reviewed computerized medical records and collected demographic data, preoperative laboratories, and treatment information. Additionally, preoperative neuroimaging findings, including the location, size, and other characteristics of the BA, were obtained.

Operational definition of certain variables was performed. Clinical sepsis statuses were defined according to study of prior studies [19, 20]. Systemic inflammatory response syndrome (SIRS) was defined as “the presence of at least two of the following criteria: fever > 38.0 °C or hypothermia < 36.0 °C, tachycardia > 90 beats/min, tachypnea > 20 breaths/min, and leukocytosis > 12 × 109/l or leukopenia < 4 × 10^9^/l,” while sepsis was defined as a documented infection in addition to SIRS. Severe sepsis was defined as sepsis in addition to at least one of the following, without any additional comorbidity or therapeutic explanation: “Glasgow coma scale ≤ 14; PaO2 ≤ 9.75 kPa; oxygen saturation ≤ 92%; PaO2/FiO2 ≤ 250; pH ≤ 7.3; lactate ≥ 2.5 mmol/l; creatinine ≥ 177 μmol/L; 100% increase in creatinine in patients with known kidney disease. oliguria (≤ 30 mL/h in ≥ 3 h or ≤ 0.7 l/24 h), prothrombin time (≤ 0.6), platelets (≤ 100*109/l), bilirubin (≥ 43 μmol/L), paralytic ileus, systolic blood pressure (≤ 90 mm Hg) or a systolic blood pressure decrease of at least 40 mm Hg from baseline.” Septic shock was defined as sepsis in combination with a systolic blood pressure of ≤ 90 mmHg or a systolic blood pressure decrease of ≥ 40 mmHg from the baseline, despite the use of vasopressor agents or adequate fluid resuscitation. Additionally, the criteria for multiple organ dysfunction syndrome were established as the presence of ≥ 2 organ failures [20–22].

Bandemia was defined as the presence of 10% or more band cells in peripheral blood smears [23], whereas the neutrophil-to-lymphocyte ratio (NLR) was the ratio of neutrophil and lymphocyte counts determined in peripheral blood and divided by a cutoff of 5 [24]. In addition, hypoalbuminemia was defined as a serum albumin level of less than 3.5 g/dL [25].

The diagnosis of BA without growth culture was determined based on a combination of neuroimaging findings that were compatible with BA, including intraoperative purulent findings and ring-enhancing lesions. Moreover, pathological findings verified an abscess even though cultures were negative.

The follow-up information was obtained until December 31, 2024. The period of survival was calculated from the date of operation to last follow-up date or death date. The last clinical status of the last follow-up comprised either censoring (still alive) or mortality. The primary source of follow-up data was patient visits to outpatient clinics. Furthermore, telephone interviews were conducted with patients (or their caregivers) who were unable to visit the hospital.

### Statistical analysis

Sample size calculation was performed with log-rank test formula by Freedman et al. [26] The hazard ratio (HR) of significant predictors in the study conducted by Sahin et al. ranged from 2.10 to 6.02. Consequently, a minimum sample size of 16–64 was required [27].

Descriptive statistics were utilized to elucidate the demographics of the current cohort. Specifically, the continuous variables were displayed by the mean and standard deviation (SD), while the categorical variables were reported as percentages.

For survival analysis, the total dataset was randomly divided into the train and test datasets in a 70:30 ratio for survival analysis. Model development was done using the train dataset, while the test dataset was used for validation. Therefore, Cox regression analysis was used to determine prognostic variables using the train dataset. All variables were screened by univariate analysis and variables that had *p*-value less than 0.1 as candidate variable. Therefore, several candidate variables were analyzed using multivariable analysis. In detail, backward stepwise produce was performed, and the final prediction model was selected by the lowest Akaike information criterion (AIC). Additionally, we used variance inflation factors (VIF) to evaluate multicollinearity among the candidate predictors. An acceptable degree of multicollinearity was indicated by VIF values < 5 for every variable included in the final model [28].

Then, the Schoenfeld residuals were checked against the Cox proportional hazards model’s assumption that a *p*-value of less than 0.05 indicates that the model does not meet the proportional hazards assumption. Furthermore, the Kaplan–Meier method was used to build survival curves. When comparing dichotomous characteristics between groups, the log-rank test was employed to estimate the *p*-value for survival.

### Nomogram development

The independent predictors influencing mortality were used to build the prediction nomogram based on the methodology established by Zhang et al. [29] To validate using both train dataset (apparent validation) and the test dataset, the nomogram’s performance was assessed using Harrell’s concordance index (C-index), and calibration plots for 1-year overall survival probability were displayed to compare predicted and observed survival after 100 times bootstrap resampling. In addition, cumulative case/dynamic control survival receiver operating characteristic (ROC) curves with area under the curve (AUC) were performed for estimate performance using test dataset. In terms of ROC curves, cumulative cases were those that died before the attended time point, whereas dynamic controls were those who survived until the attended time point [30, 31]. The time points (12 to 120 months at 12-month intervals) used for the time-dependent ROC analysis were chosen for their clinical significance on the long-term prognosis of patients with brain abscesses. The discriminative performance of the model over short-, intermediate-, and long-term follow-up can be visualized using these intervals. Finally, a dynamic nomogram was created that computes and displays changing survival curves when personal variables change. The statistical analysis was conducted out using the R version 4.4.0 software, while dynamic nomogram was created by DynNom package (R Foundation, Vienna, Austria).

### Ethical considerations

The study was conducted in accordance with the Declaration of Helsinki (as revised in 2013). A human research ethics committee approved the present study (REC 66–433-10–1). Because it was a retrospective analysis, the current study did not require patients’ informed consent. However, the identity numbers of patients were encoded before the analysis.

## Results

As the result, Table 1 presents the clinical features of the 205 patients with brain abscesses. There was a slight male predominance at 54.6%, with a mean age of 40.96 (± 21.90) years. Common underlying conditions were sinusitis, cyanotic heart disease, and diabetes mellitus. SIRS, sepsis, severe sepsis, septic shock, and multiorgan dysfunction were identified in 8.3%, 27.3%, 10.2%, 2.0%, and 5.4% of the patients, respectively. Furthermore, 11.2% of the samples yielded positive hemoculture results. Gram-positive, gram-negative, and anaerobes were detected in 31.7%, 12.2%, and 10.7% of cases, respectively, for microorganism identification. Fungal and tuberculous BA were identified in 8.8% and 3.4% of cases.

**Table 1:** Demographic data of total dataset (*N* = 205)

Factor	Total dataset	Train dataset	Test dataset	*p*-value
	***N*** (%)	***N*** (%)	***N*** (%)	
**Gender**				0.83
Male	112 (54.6)	78 (54.2)	34 (55.7)	
Female	93 (45.4)	66 (45.8)	27 (44.3)	
**Mean age—year (SD)**	40.96 (21.90)	42.20 (21.19)	38.04 (23.40)	0.21
**Age group—year**				0.53
< 60	166 (81.0)	115 (79.9)	51 (83.6)	
≥ = 60	39 (19.0)	29 (20.1)	10 (16.4)	
**Glasgow coma score on admission**				0.93
13–15	165 (80.5)	116 (80.6)	49 (80.3)	
9–12	25 (12.2)	18 (12.5)	7 (11.5)	
3–8	15 (7.3)	10 (6.9)	5 (8.2)	
**Karnofsky performance status**				0.42
< 80	182 (88.8)	62 (43.1)	30 (49.2)	
> = 80	23 (11.2)	82 (56.9)	31 (50.8)	
**Underlying disease**				
Sinusitis	23 (11.2)	16 (11.1)	7 (11.5)	0.94
Cyanotic heart disease	17 (8.3)	11 (7.6)	6 (9.8)	0.60
Diabetes mellitus	16 (7.8)	9 (6.3)	7 (11.5)	0.20
Pneumonia	14 (6.8)	12 (8.3)	2 (3.3)	0.19
Otitis media	13 (6.3)	11 (7.6)	2 (3.3)	0.24
HIV infection	10 (4.9)	7 (4.9)	3 (4.9)	0.98
Hematologic carcinoma	9 (4.4)	8 (5.6)	1 (1.6)	0.21
Hypertension	9 (4.4)	7 (4.9)	2 (3.3)	0.61
Meningitis	9 (4.4)	6 (4.2)	3 (4.9)	0.81
Dental caries	8 (3.9)	4 (2.8)	4 (6.6)	0.20
Febrile neutropenia	8 (3.9)	8 (5.6)	0 (0)	0.06
Prolong steroid usage	7 (3.4)	7 (4.9)	0 (0)	0.08
Cerebrovascular disease	3 (1.5)	2 (1.4)	1 (1.6)	0.89
Cirrhosis	3 (1.5)	2 (1.4)	1 (1.6)	0.89
**Hemoculture**				0.57
No growth	182 (88.8)	129 (89.6)	53 (86.9)	
Positive	23 (11.2)	15 (10.4)	8 (13.1)	
**Sepsis status**				0.76
No sepsis	96 (46.8)	69 (47.9)	27 (44.3)	
SIRS	17 (8.3)	14 (9.7)	3 (4.9)	
Sepsis	56 (27.3)	37 (25.7)	19 (31.1)	
Severe sepsis	21 (10.2)	13 (9.0)	8 (13.1)	
Septic shock	4 (2.0)	3 (2.1)	1 (1.6)	
Multiorgan dysfunction	11 (5.4)	8 (5.6)	3 (4.9)	
**Organism from pus culture/pathological result**				0.87
No growth	62 (30.2)	45 (31.3)	17 (27.9)	
Gram positive	65 (31.7)	48 (33.3)	17 (27.9)	
Gram negative	25 (12.2)	16 (11.1)	9 (14.8)	
Anaerobes	22 (10.7)	13 (9.0)	9 (14.8)	
Fungus	18 (8.8)	13 (9.0)	5 (8.2)	
Tuberculosis	7 (3.4)	5 (3.5)	2 (3.3)	
Toxoplasmosis	6 (2.9)	4 (2.8)	2 (3.3)	
**Brain abscess location**				
Frontal lobe	115 (56.1)	79 (54.9)	36 (59.0)	0.58
Temporal lobe	60 (29.3)	40 (27.8)	20 (32.8)	0.47
Parietal lobe	83 (40.5)	59 (41.0)	24 (39.3)	0.82
Occipital lobe	36 (17.6)	29 (20.1)	7 (11.5)	0.13
Basal ganglion/thalamus	21 (10.2)	15 (10.4)	6 (9.8)	0.90
Cerebellum	23 (11.2)	15 (10.4)	8 (13.1)	0.57
Sellar/parasellar area	5 (2.4)	4 (2.8)	1 (1.6)	0.62
**Mean maximum diameter of abscess—cm (SD)**	3.29 (1.66)	3.48 (1.64)	3.18 (1.69)	0.23
**Maximum diameter group—cm**				0.43
< 3	89 (43.4)	60 (41.7)	29 (47.5)	
≥ 3	116 (56.6)	84 (58.3)	32 (52.5)	
**Number of brain abscess**				0.93
Single	142 (69.3)	100 (69.4)	42 (68.9)	
Multiple	63 (30.7)	44 (30.6)	19 (31.1)	
**Venous thrombosis**	6 (2.9)	4 (2.8)	2 (3.3)	0.83
**Leptomeningeal enhancement**	55 (26.8)	41 (28.5)	14 (23.0)	0.41
**Ventriculitis**	35 (17.1)	25 (17.4)	10 (16.4)	0.86
**Intraventricular rupture**	17 (8.3)	13 (9.0)	4 (6.7)	0.57
**Mean midline shift—mm**	3.48 (2.93)	4 (2.8)	1 (1.6)	0.62
**Midline shift—mm**				0.29
< 5	137 (66.8)	93 (64.6)	44 (72.1)	
≥ 5	68 (33.2)	51 (35.4)	17 (27.9)	
**Obliterated basal cistern**	60 (29.3)	46 (31.9)	14 (23.0)	0.19
**Laboratory**				
Anemia	32 (15.6)	24 (16.7)	8 (13.1)	0.52
Bandemia (10% or more)	4 (2.0)	4 (2.8)	0 (0)	0.18
Thrombocytopenia	9 (4.4)	5 (3.5)	4 (6.6)	0.32
Coagulopathy	9 (4.4)	7 (4.9)	2 (3.3)	0.61
Neutrophil-to-lymphocyte ratio				0.52
< 5	114 (55.6)	78 (54.2)	36 (59.0)	
≥ 5	91 (44.4)	66 (45.8)	25 (41.0)	
Hypoalbuminemia	85 (41.5)	62 (43.1)	23 (37.7)	0.47
**Type of surgery**				0.53
Burr hole with aspiration	104 (50.7)	68 (47.2)	36 (59.0)	
Craniotomy with abscess excision	96 (46.8)	72 (50.0)	24 (39.3)	
Endoscopic endonasal transsphenoidal surgery	5 (2.4)	4 (2.8)	1 (1.6)	
**Time follow-up point**				
Survival at 1-year follow-up	108 (52.7)	72 (50.0)	36 (59.0)	0.23
Survival at 2-year follow-up	89 (43.4)	62 (43.1)	27 (44.3)	0.87
Survival at 5-year follow-up	55 (26.8)	37 (25.7)	18 (29.5)	0.57

BA is the most common location in the frontal lobe, accounting for 56.1% of cases, whereas occipital BA was observed in 17.6% of cases. According to the present study, the average preoperative BA diameter was 3.29 (± 1.66) cm, and 30.7% of cases had multiple BA. According to preoperative laboratory tests, 44.4% of patients had an NLR of 5 or above, and 2% of patients had bandemia. Moreover, serum albumin level below 3.5 mg/dL was found in 41.5%.

The endoscopic endonasal transsphenoidal surgery was performed to treatment in 2.4% of the present cohort. The surgical approach was used in select cases where lesions involving the sellar or parasellar area typically resulted from invasive fungal sinusitis.

### Survival analysis

**Fig. 1:**
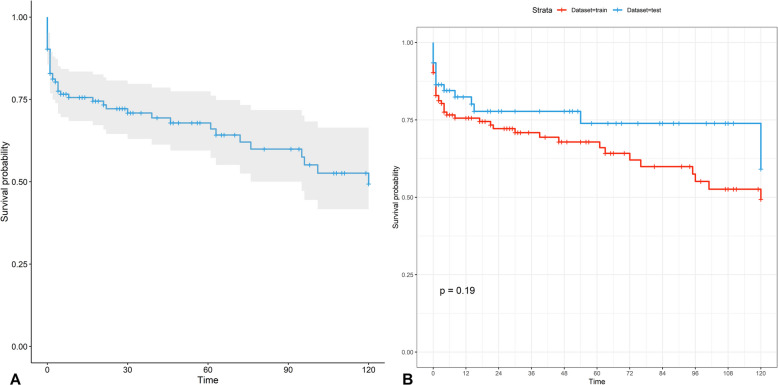
Kaplan–Meier curves of patients with primary brain abscess. **A** Overall survival. **B** Kaplan–Meier curves of training dataset and test dataset (*p*-value of log-rank test = 0.19)

The mean follow-up time was 41.66 (± 57.19) months, and the maximum follow-up period was 120 months. As a result, 1-year, 2-year, and 5-year survival probabilities were 0.77 (95% confidence interval (*CI*) 0.71–0.83), 0.73 (95% *CI* 0.67–0.80), and 0.69 (95% *CI* 0.62–0.77), respectively. Additionally, the median overall survival time was determined; however, the estimated median survival was not achieved from the entire dataset, and overall survival curve is demonstrated in Fig. 1A. Train and test datasets were generated from randomly splitting with a ratio of 70:30; overall survival curves from both datasets did not differ (*p*-value of log-rank test, 0.19), as shown in Fig. 1B. Moreover, Table 2 shows 1-year, 2-year, and 5-year survival probability according to microorganism. The 5-year survival probability for gram-positive bacteria and gram-positive bacteria was 0.72 (95% *CI* 0.60–0.86) and 0.86 (95% *CI* 0.74–1.00), respectively. Whereas tuberculous BA had a 5-year survival probability of 0.85 (95% *CI* 0.63–1.00), anaerobic bacterial survival probability was 0.81 (95% *CI* 0.63–1.00). Furthermore, fungal BA showed a poor prognosis, with a 5-year survival probability of 0.19 (95% *CI* 0.04–0.88).

**Table 2:** One-year, two-year, and five-year survival probabilities according to microorganism (*N* = 205)

Group	1-year survival probability (95% *CI*)	2-year survival probability (95% *CI*)	5-year survival probability (95% *CI*)
Overall	0.77 (0.71–0.83)	0.73 (0.67–0.80)	0.69 (0.62–0.77)
No growth	0.71 (0.60–0.84)	0.67 (0.54–0.81)	0.67 (0.54–0.81)
Gram-positive bacteria	0.85 (0.76–0.94)	0.82 (0.73–0.93)	0.72 (0.60–0.86)
Gram-negative bacteria	0.86 (0.74–1.00)	0.86 (0.74–1.00)	0.86 (0.74–1.00)
Anaerobic bacteria	0.89 (0.76–1.00)	0.81 (0.63–1.00)	0.81 (0.63–1.00)
Toxoplasmosis	0.83 (0.58–1.00)	0.83 (0.58–1.00)	0.83 (0.58–1.00)
Tuberculosis	0.85 (0.63–1.00)	0.85 (0.63–1.00)	0.85 (0.63–1.00)
Fungus	0.38 (0.20–0.73)	0.19 (0.04–0.88)	0.19 (0.04–0.88)

**Fig. 2:**
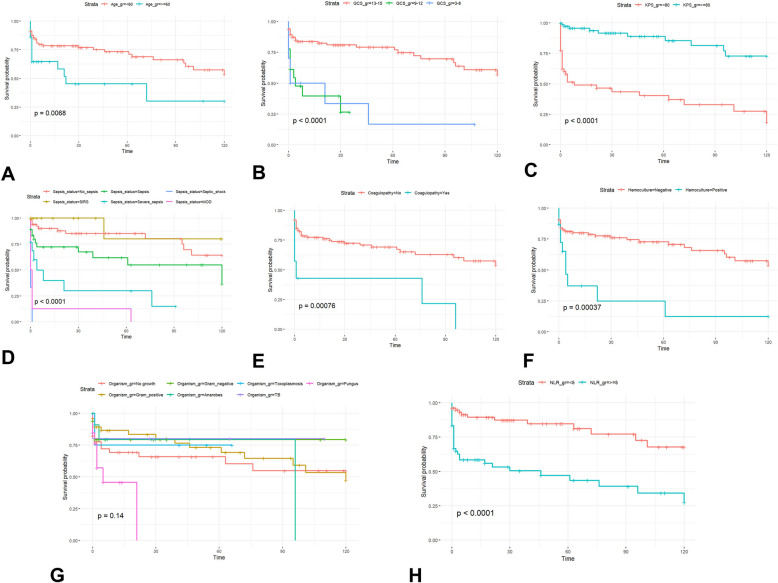
Kaplan–Meier curves by clinical, imaging, and laboratory variables. **A** Age group. **B** Glasgow Coma Scale score (GCS) group. **C** Karnofsky performance status (KPS) group. **D** Sepsis status. **E** Coagulopathy. **F** Hemoculture result. **G** Organism group. **H** Neutrophil-to-lymphocyte ratio (NLR) group. **I** Bandemia. **J** Hypoalbuminemia. **K** Occipital abscess

From univariate analysis, the candidate variables with *p*-value 0.1 or less included age group, Glasgow Coma Scale score group, Karnofsky performance status (KPS) group, positive result from hemoculture, sepsis status, preoperative coagulopathy, microorganisms, NLR, bandemia, hypoalbuminemia, and occipital BA. In addition, survival curves by candidate predictors are displayed in Fig. 2. Hence, the final prediction model that had the lowest AIC value comprised the age group, KPS group, positive result from hemoculture, preoperative coagulopathy, NLR, and occipital BA, as shown in Table 3. The *p*-value of the global Schoenfeld test was 0.16; therefore, the final model was assessed for model performance using both apparent validation and test dataset validation. Moreover, multicollinearity was assessed using the VIF, and all predictors had VIFs below 5.

**Table 3:** Factor associated with prognosis from the training dataset (*N* = 144)

Factor	Univariate analysis		Multivariable analysis	
	**Hazard ratio** **(95% *CI*)**	***p*-value**	**Hazard ratio** **(95% *CI*)**	***p*-value**
**Gender**				
Male	Ref			
Female	0.77 (0.43–1.39)	0.42		
**Age group**				
< 60	Ref		Ref	
≥ 60	2.36 (1.26–4.46)	0.008	2.70 (1.39–5.25)	0.003
**Glasgow coma score on admission**				
13–15	Ref			
9–12	4.73 (2.26–9.87)	< 0.001		
3–8	4.69 (2.03–10.83))	< 0.001		
**Karnofsky performance status**				
< 80	Ref		Ref	
> = 80	0.14 (0.07–0.28)	< 0.001	0.15 (0.07–0.32)	< 0.001
Sinusitis*	1.58 (0.66–3.75)	0.30		
Cyanotic heart disease*	1.30 (0.51–3.28)	0.68		
Diabetes mellitus*	1.11 (0.34–3.58)	0.98		
Pneumonia*	1.37 (0.54–0.34)	0.52		
Otitis media*	0.64 (0.16–2.67)	0.54		
HIV infection*	1.76 (0.54–5.73)	0.37		
Hematologic carcinoma*	0.96 (0.23–3.99)	1.01		
Hypertension*	0.47 (0.06–3.43)	0.56		
Meningitis*	2.16 (0.77–6.03)	0.14		
Dental caries*	2.43 (0.58–10.07)	0.20		
Febrile neutropenia*	3.75 (0.75–8.50)	0.12		
Prolong steroid usage*	0.38 (0.05–2.74)	0.33		
Cerebrovascular disease*	1.45 (0.20–10.61)	0.73		
Cirrhosis*	3.72 (0.89–15.50)	0.07		
**Hemoculture**				
No growth	Ref		Ref	
Positive	3.43 (1.69–7.00)	< 0.001	1.99 (0.93–13.02)	0.001
**Sepsis status**				
No sepsis	Ref			
SIRS	0.36 (0.05–2.78)	0.33		
Sepsis	2.44 (1.11–5.35)	0.03		
Severe sepsis	6.70 (2.77–16.23)	< 0.001		
Septic shock	34.57 (9.01–132.70)	< 0.001		
Multiorgan dysfunction	18.72 (7.35–47.69)	< 0.001		
**Organism**				
No growth	Ref			
Gram positive	0.76 (0.37–1.53)	0.44		
Gram negative	0.66 (0.19–2.29)	0.52		
Anaerobes	0.81 (0.24–2.81)	0.74		
Fungus	2.65 (1.05–6.65)	0.04		
Tuberculosis	0.51 (0.07–3.87)	0.52		
Toxoplasmosis	0.69 (0.09–5.23)	0.72		
Frontal abscess*	0.77 (0.43–1.37)	0.46		
Temporal abscess*	1.08 (0.58–2.04)	0.87		
Parietal abscess*	0.85 (0.47–1.53)	0.69		
Occipital abscess*	0.42 (0.18–0.99)	0.05	0.34 (0.13–0.87)	0.02
Basal ganglion/thalamic abscess*	1.18 (0.47–3.00)	0.78		
Cerebellar abscess*	0.97 (0.38–2.48)	1.02		
**Maximum diameter group—cm**				
< 3	Ref			
≥ 3	1.00 (0.84–1.18)	1.09		
**Maximum diameter group—cm**				
< 3	Ref			
≥ 3	1.00 (0.84–1.18)	1.09		
**Number of brain abscess**				
Single	Ref			
Multiple	0.86 (0.46–1.63)	0.76		
Venous thrombosis*	2.15 (0.52–8.98)	0.32		
Leptomeningeal enhancement*	1.52 (0.84–2.77)	0.23		
Ventriculitis*	1.62 (0.82–3.19)	0.27		
Intraventricular rupture*	1.58 (0.67–3.74)	0.35		
**Midline shift—mm**				
< 5	Ref			
≥ 5	1.02 (0.96–1.08)	0.64		
Obliterated basal cistern*	1.06 (0.58–1.95)	0.86		
Anemia*	1.86 (0.94–3.66)	0.07		
**Bandemia**				
< 10%	Ref			
≥ 10%	9.69 (3.39–27.78)	< 0.001		
Thrombocytopenia*	0.85 (0.12–6.22)	0.90		
Coagulopathy*	4.18 (1.76–9.94)	0.001	4.98 (1.91–13.02)	0.001
**Neutrophil-to-lymphocyte ratio**				
< 5	Ref		Ref	
≥ 5	4.19 (2.20–7.99)	< 0.001	3.96 (1.94–8.09)	< 0.001
Hypoalbuminemia*	1.97 (1.10–3.54)	0.02		
**Type of surgery**				
Burr hole with aspiration	Ref			
Craniotomy with abscess excision	1.43 (0.78–2.60)	0.23		
Endoscopic endonasal transsphenoidal surgery	1.05 (0.14–7.89)	0.96		

*Data show only the “yes group” while the reference group (no group) are hidden

**Fig. 3:**
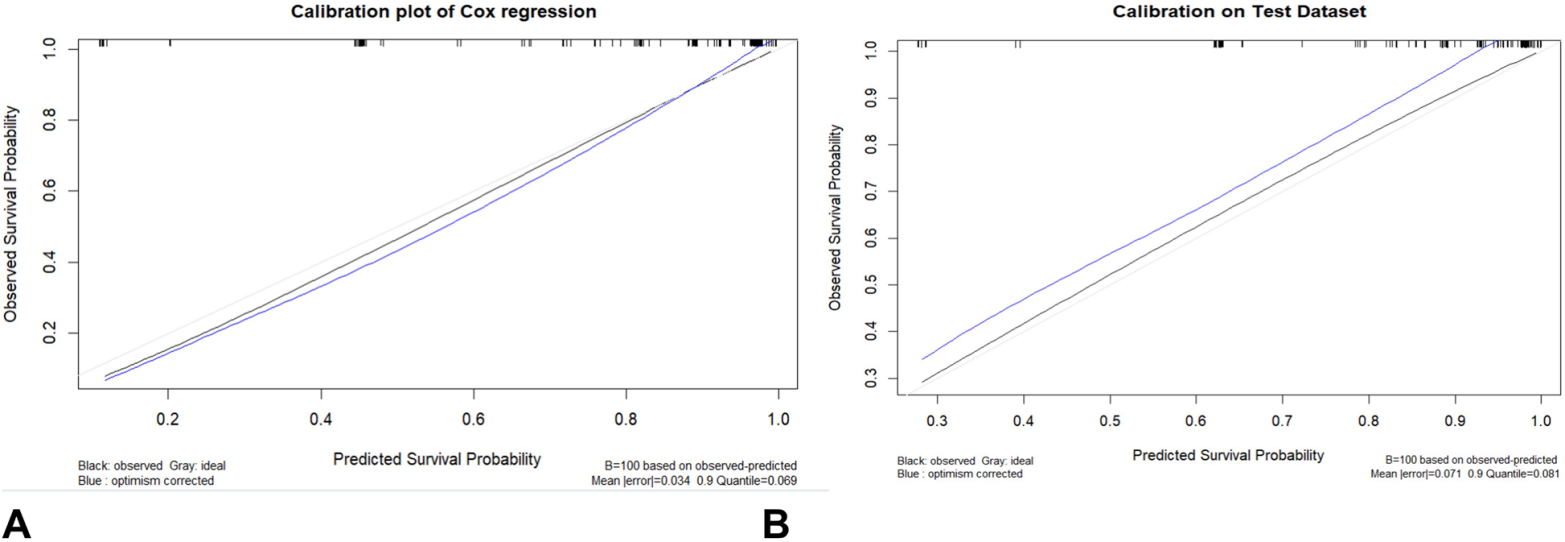
Calibration plot with 100 times bootstrap resampling. **A** Train dataset. **B** Test dataset

**Fig. 4:**
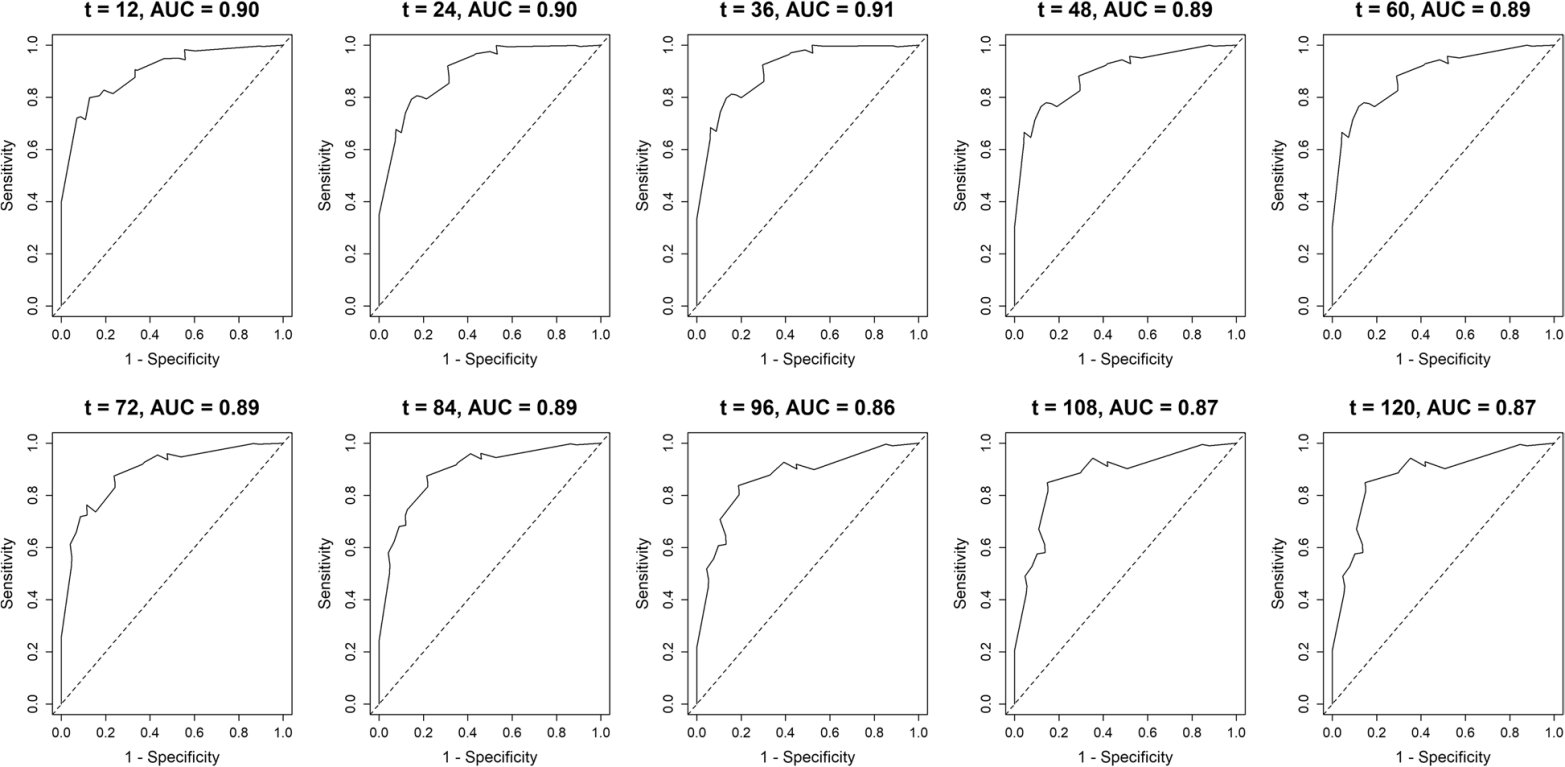
Time-dependent ROC curves with area under the curve (AUC) for primary brain abscess prognostication at various specific time points (t-month)

For the apparent validation examined in the model performance by the train dataset, C-index was 0.855, and the bootstrapped calibration plot shows that the prediction of the model is quite close to the ideal 45° ideal line, as shown in Fig. 3A. The C-index of model validation computed using the test dataset was 0.701, and Fig. 3B depicts a bootstrapped calibration curve indicating that the projected outcome corresponds satisfactorily to the actual performance. Furthermore, Fig. 4 illustrates the cumulative case/dynamic control survival ROC curves for a variety of specific time intervals, with an AUC that ranged from 0.86 to 0.93.

**Fig. 5:**
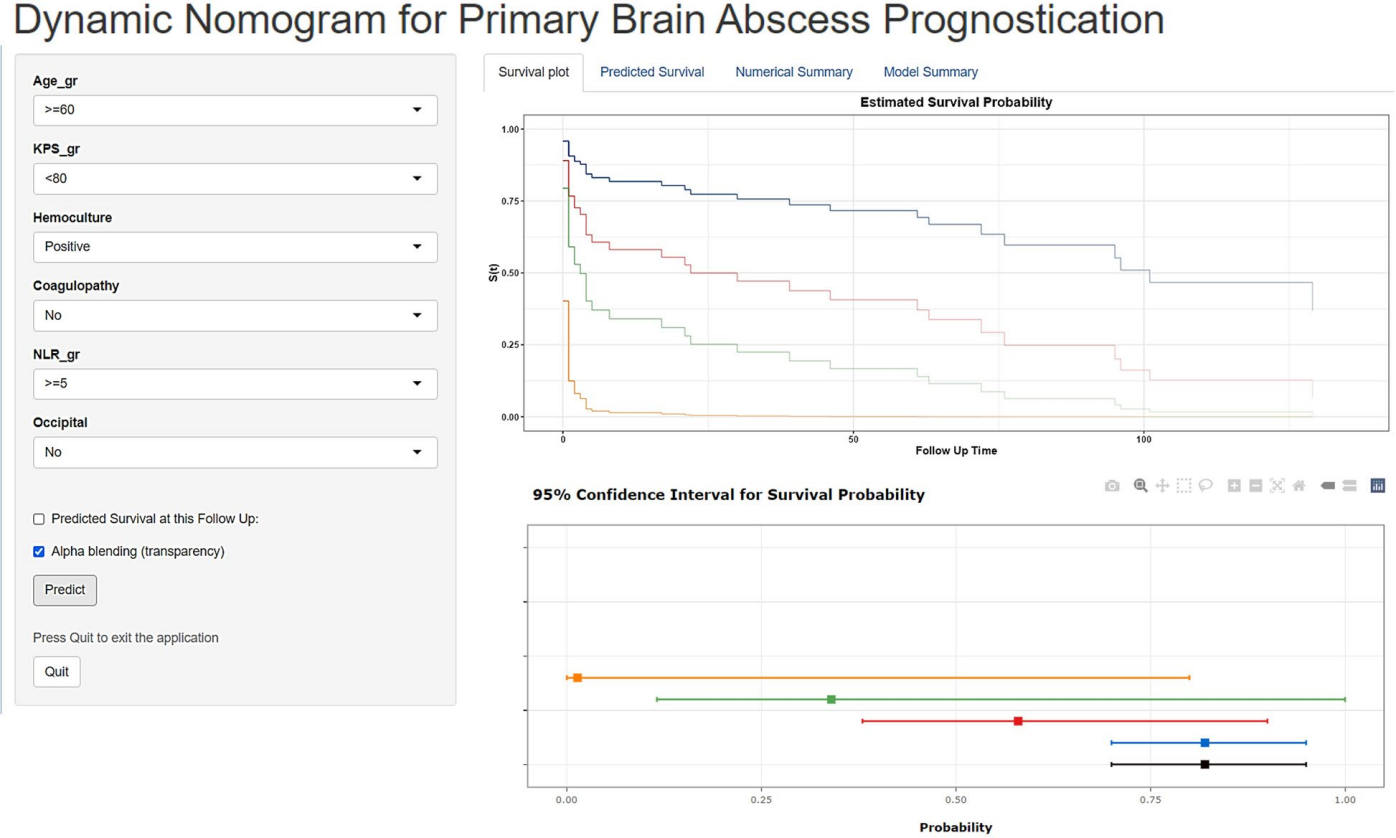
Dynamic nomogram predicting prognosis of patients with brain abscess

The final prediction model was provided as a dynamic nomogram software that can run the R script (https://github.com/thara7640/DynNom_BA) on each laptop and use the program, as illustrated in Fig. 5. As a result, when the predictors change, the survival curve and projected survival probability will also change.

## Discussion

In the present study, the observed 5-year survival probability was 69%, indicating a case fatality rate that remains comparatively high. Prior systematic reviews and meta-analyses have documented a broad spectrum of mortality rates in individuals with BA, often ranging from 10 to 40% [1]. Bodilsen et al. observed that overall mortality during follow-up was 47%, with a 1-year death probability of 21% [5, 9]. More recently, Sahin et al. reported survival probabilities of 91.9% at 42 days and 86.1% at 180 days, suggesting improved short-term outcomes in certain cohorts [27]. There are several possible explanations for this discrepancy. First, the study was conducted in a single tertiary center in a resource-limited region, which could be attributed to delayed referral and treatment initiation. Second, our cohort included a higher proportion of severe and atypical infections, such as fungal and tuberculous BA, which are known to have poor prognoses. Third, comorbidities such cyanotic heart disease, diabetes, and sepsis were common in our cohort and may have contributed to higher mortality.

Age 60 years or older and KPS 80 or higher were clinical predictors that were statistically linked to prognosis in this study, while occipital placement was an imaging prediction that was linked to a favorable prognosis. These results are in concordance with previous studies. Bodilsen et al. used the national population-based medical registries to study the prognosis of patients with BA and observed that older age, comorbidity index, and immune-compromised status were associated significantly with mortality [5, 9]. These clinical predictors are associated with a prolonged survival time, which may be attributed to a potential deterioration of physiological reserves, a weakened immune system, and a decrease in functional capacity in patients who are elderly and have a low KPS [32, 33]. The present study demonstrated that occipital BA was associated with a more favorable prognosis, which could be owing to its ease of access [34].

In laboratory predictors, the presence of bacteria or other microorganisms in the circulation, associated with a poor prognosis in the present investigation, indicates a poor prognosis. Moreover, bandemia and high NLR were significantly related to poor prognosis. This is perhaps explained by these indicators reflecting high pathogen load and overwhelmed degree of infection, so compromising the body’s capacity for control of the disease [35].

Various prognostic factors were used to estimate the prognosis of BA patients using a nomogram. The apparent validation of the nomogram had a C-index of 0.855, while the C-index of model validation computed using the test dataset was 0.701. As a result, the C-index dropped which could indicate overfitting. To reduce overfitting, external validation in multicenter or prospective cohorts is required, allowing to enhance generalizability [12, 35]. Nonetheless, a C-index value greater than 0.7 suggests a good fit between the observed outcomes and the predictions [36]. As a result, the nomogram may be a valuable tool for personalized risk assessments, but the difficulty is that an individual’s prognosis may alter due to modifiable variables. Dynamic nomograms offer several advantages over traditional static nomograms, particularly in terms of interactivity, flexibility, and user accessibility [12, 37]. Because traditional nomograms are often shown as 2-dimensional graphic figures with rating scales that might be inaccurately assigned scores, dynamic nomograms are typically offered as web-based tools that allow users to enter patient-specific information and receive quick, individualized forecasts. This interaction improves the user experience and enables real-time decision-making [15].

To the authors’ knowledge, this was the first study to employ a dynamic nomogram for prognostication in BA patients. Nevertheless, it was acknowledged that the current investigation was subject to certain limitations. Information bias has been generated by the study’s retrospective methodology, while generalizability problem and selection bias were concern from single-center setting [38]. The problem of generalizability may be resolved through multicenter research in the future, which could broaden the study population, facilitate the nomogram’s further refinement, and enhance its clinical utility in a variety of populations [39]. While the predictive model in the present study included key clinical and laboratory factors that are known to be associated with adverse outcomes in BA, we recognize that many of these factors—including age, functional status, inflammatory markers, and coagulopathy—are non-specific and may not be able to fully capture the unique pathophysiological processes of BA. Therefore, the model may have limited ability to differentiate risk among clinically heterogeneous patient subgroups. Future models could be significantly strengthened by integrating pathogen-specific characteristics (such as virulence factors, antimicrobial resistance profiles), abscess morphology, molecular biomarkers of immune response, and advanced neuroimaging features (such as diffusion-weighted imaging patterns or MR spectroscopy).[2, 27, 39] These enhancements may increase the pathophysiological specificity of prognostic models, allowing for personalized, clinically actionable decision-making in BA management [40].

## Conclusion

This study developed and validated a robust prognostic model for long-term survival in patients with primary brain abscess. Key predictors of survival included age, KPS, hemoculture results, preoperative coagulopathy, NLR, bandemia, and occipital BA. The predictive model demonstrated high accuracy and reliability, with a dynamic nomogram offering a user-friendly tool for individualized survival predictions. Future research should focus on external validation and further refinement of the model to ensure its generalizability across diverse populations.

## Data Availability

The dataset provided for this study is available on request to the corresponding author.

## Abbreviations

AIC: Akaike information criterion
AUC: Area under the curve
BA: Brain abscess
C-index: Harrell’s concordance index
CPT: Clinical prediction tool
GCS: Glasgow Coma Scale
HR: Hazard ratio
KPS: Karnofsky performance status
NLR: Neutrophil-to-lymphocyte ratio
ROC: Receiver operating characteristic
SD: Standard deviation
SIRS: Systemic inflammatory response syndrome
